# Redetermination of the cubic struvite analogue Cs[Mg(OH_2_)_6_](AsO_4_)

**DOI:** 10.1107/S1600536808043171

**Published:** 2008-12-20

**Authors:** Matthias Weil

**Affiliations:** a Institute for Chemical Technologies and Analytics, Division of Structural Chemistry, Vienna University of Technology, Getreidemarkt 9/164-SC, A-1060 Vienna, Austria

## Abstract

In contrast to the previous refinement from photographic data [Ferrari *et al.* (1955 ▶). *Gazz. Chim. Ital.* **84**, 169–174], the present redetermination of the title compound, caesium hexa­aqua­magnesium arsenate(V), revealed the Cs atom to be on Wyckoff position 4*d* instead of Wyckoff position 4*b* of space group *F* 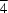
3*m*. The structure can be derived from the halite structure. The centres of the complex [Mg(OH_2_)_6_] octa­hedra and the AsO_4_ tetra­hedra (both with 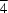
3*m* symmetry) are on the respective Na and Cl positions. The building units are connected to each other by O—H⋯O hydrogen bonds. The Cs^+^ cations (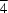
3*m* symmetry) are located in the voids of this arrangement and exhibit a regular cubocta­hedral 12-coordination to the O atoms of the water mol­ecules. The O atom bonded to As has 2*mm* site symmetry (Wyckoff position 24*f*) and the water-mol­ecule O atom has *m* site symmetry (Wyckoff position 48*h*).

## Related literature

The crystal structure of struvite, NH_4_[Mg(OH_2_)_6_](PO_4_), was reported by Whitaker & Jeffery (1970*a* ▶,*b* ▶). Crystal growth of struvite-type compounds using the gel diffusion technique was reported by Banks *et al.* (1975 ▶). For isotypic structures, see: Carver *et al.* (2006 ▶) for Cs[Fe(OH_2_)_6_](PO_4_) and Massa *et al.* (2003 ▶) for the cubic form of dimorphic Cs[Mg(OH_2_)_6_](PO_4_). Isotypic struvite-type phases as well as analogues were recently surveyed by Weil (2008 ▶).

## Experimental

### 

#### Crystal data

Cs[Mg(OH_2_)_6_](AsO_4_)
*M* *_r_* = 404.24
Cubic, 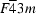

*a* = 10.1609 (5) Å
*V* = 1049.05 (9) Å^3^
*Z* = 4
Mo *K*α radiation
μ = 6.75 mm^−1^
*T* = 293 (2) K
0.18 × 0.18 × 0.18 mm

#### Data collection

Enraf–Nonius CAD-4 diffractometer
Absorption correction: integration (*SHELXTL*; Sheldrick, 2008 ▶) *T*_min_ = 0.341, *T*_max_ = 0.382
3332 measured reflections
208 independent reflections
207 reflections with *I* > 2σ(*I*)
*R*_int_ = 0.037
3 standard reflections frequency: 200 min intensity decay: none

#### Refinement

*R*[*F* ^2^ > 2σ(*F* ^2^)] = 0.016
*wR*(*F* ^2^) = 0.038
*S* = 1.14
208 reflections
15 parameters
1 restraint
H atoms treated by a mixture of independent and constrained refinement
Δρ_max_ = 0.50 e Å^−3^
Δρ_min_ = −0.48 e Å^−3^
Absolute structure: Flack (1983 ▶), 90 Friedel pairs
Flack parameter: 0.01 (4)

### 

Data collection: *CAD-4 Software* (Enraf–Nonius, 1989 ▶); cell refinement: *CAD-4 Software*; data reduction: *HELENA* implemented in *PLATON* (Spek, 2003 ▶); method used to solve structure: coordinates taken from an isotypic compound; program(s) used to refine structure: *SHELXL97* (Sheldrick, 2008 ▶); molecular graphics: *ATOMS* (Dowty, 2004 ▶); software used to prepare material for publication: *SHELXL97*.

## Supplementary Material

Crystal structure: contains datablocks I, global. DOI: 10.1107/S1600536808043171/hb2885sup1.cif

Structure factors: contains datablocks I. DOI: 10.1107/S1600536808043171/hb2885Isup2.hkl

Additional supplementary materials:  crystallographic information; 3D view; checkCIF report

## Figures and Tables

**Table 1:** Selected bond lengths (Å)

Cs1—O1^i^	3.6239 (5)
Mg1—O1	2.064 (3)
As1—O2	1.681 (3)

Symmetry code: (i) 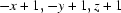.

**Table 2:** Hydrogen-bond geometry (Å, °)

*D*—H⋯*A*	*D*—H	H⋯*A*	*D*⋯*A*	*D*—H⋯*A*
O1—H1⋯O2^ii^	0.79 (2)	1.86 (2)	2.650 (2)	176 (4)

Symmetry code: (ii) 
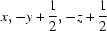
.

**Fig. 1.**
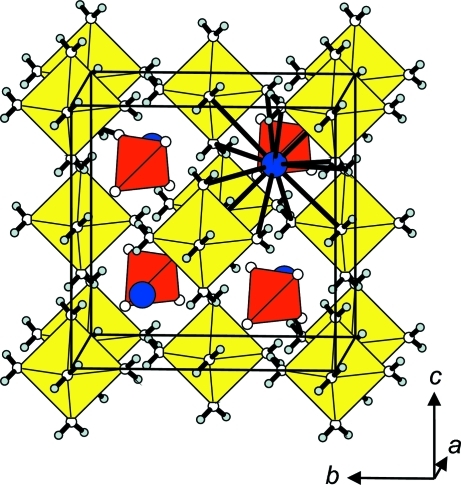
The crystal structure of Cs[Mg(H2O)6](AsO4) projected approximately along [001]. [Mg(OH2)6] octahedra are yellow, PO4 tetrahedra are red, Cs atoms are blue O atoms are white and H atoms are grey. For one of the Cs+ cations the Cs–O bonds are indicated.

## supplementary crystallographic information

### Comment

Numerous compounds with general formula *A*[*B*(OH_2_)_6_]*X*O_4_, where *A* = alkali metal, NH_4_ or Tl, *B* = Mg or first row transition metal, and *X* = P or As, are known to crystallize in the orthorhombic struvite (NH_4_[Mg(OH_2_)_6_](PO_4_)) structure in space group *Pmn*2_1_ (Whitaker & Jeffrey, 1970*a*, *b*). The title compound, (I), Cs[Mg(OH_2_)_6_](AsO_4_), is an isoformular analogue of struvite, but crystallizes in the cubic crystal system. All these structures can be described in terms of closed-packed layers with different stacking sequences (Massa *et al.*, 2003). Phases that are isotypes of struvite, as well as struvite analogues were recently surveyed by Weil (2008). In comparison with the previous refinement from photographic data (Ferrari *et al.*, 1955), the present redetermination of Cs[Mg(OH_2_)_6_](AsO_4_) revealed a different location of the Cs atom, the localization of the H atom and anisotropic displacement parameters for all non H-atoms.

The structure can be described as a derivative of the NaCl structure type (Massa *et al.*, 2003). The centres of the regular complex [Mg(H_2_O)_6_] octahedra are situated on the respective Na positions, and the centres of the regular AsO_4_ tetrahedra are situated on the Cl positions (Fig. 1). Thus one [Mg(H_2_O)_6_] octahedron is surrounded by six AsO_4_ tetrahedra in an octahedral arrangement, and *vice versa*. The corresponding Mg—O and As—O distances are in the normal range (Table 1). These building units are linked *via* medium-strong hydrogen bonds (Table 2). Details and differences of the hydrogen bonding schemes in cubic, hexagonal and orthorhombic struvite-type structures were discussed in detail by Massa *et al.* (2003). The Cs^+^ cations are located in the voids of this arrangement and exhibit a regular cuboctahedral 12-coordination to the oxygen atoms of the water molecules (Table 1).

### Experimental

Colourless octahedral crystals of Cs[Mg(OH_2_)_6_](AsO_4_) with an edge-length up to 1 mm were grown by means of the gel diffusion technique, following a slightly modified procedure as that given by Banks *et al.* (1975). Aqueous solutions of 0.025 *M* MgSO_4_ and 0.02 *M* Na_4_edta (edta = ethylenediaminetetraacetate) were adjusted to pH 10 with NaOH. Commercially available gelatine foils (5 g) were dissolved in the hot resulting 100 ml solution and allowed to form a gel inside a large test tube overnight. When the gel had set, an equivalent amount of a solution of 0.025 *M* CsH_2_AsO_4_ (50 ml) was carefully poured over the gel. This solution was then adjusted to pH 9 with NaOH. The test tube was covered with parafilm and the crystal growth proceeded at the gel-liquid interface and into the gel. Crystals large enough for conventional *x*-ray analysis grew within one week at room temperature. They were separated mechanically from the gel and were washed with a water/ethanol/acetone (1/3/1) mixture.

### Refinement

The coordinates of the isotypic compound Cs[Fe(OH_2_)_6_](PO_4_) (Carver *et al.*, 2006) were taken as starting parameters. The Cs atom in the original structure determination (Ferrari *et al.*, 1955) was positioned on Wyckoff site 4*b* (1/2, 1/2, 1/2), whereas in the present refinement Cs is on position 4*d* (3/4, 3/4, 3/4). The position of the H atom was found from difference Fourier maps and was refined with a soft distance restraint of O—H = 0.85 (3) Å.

### Crystal data

**Table:**

Cs[Mg(H_2_O)_6_](AsO_4_)	*D*_x_ = 2.559 Mg m^−^^3^
*M**_r_* = 404.24	Mo *K*α radiation, λ = 0.71073 Å
Cubic, *F*43*m*	Cell parameters from 25 reflections
Hall symbol: F -4 2 3	θ = 11.4–12.7°
*a* = 10.1609 (5) Å	µ = 6.75 mm^−^^1^
*V* = 1049.05 (9) Å^3^	*T* = 293 K
*Z* = 4	Octahedron, colourless
*F*(000) = 768	0.18 × 0.18 × 0.18 mm

### Data collection

**Table:**

Enraf–Nonius CAD-4 diffractometer	207 reflections with *I* > 2σ(*I*)
Radiation source: fine-focus sealed tube	*R*_int_ = 0.037
graphite	θ_max_ = 30.9°, θ_min_ = 3.5°
ω/2θ scans	*h* = −14→14
Absorption correction: integration (*SHELXTL*; Sheldrick, 2008)	*k* = −14→14
*T*_min_ = 0.341, *T*_max_ = 0.382	*l* = −14→14
3332 measured reflections	3 standard reflections every 200 min
208 independent reflections	intensity decay: none

### Refinement

**Table:**

Refinement on *F*^2^	H atoms treated by a mixture of independent and constrained refinement
Least-squares matrix: full	*w* = 1/[σ^2^(*F*_o_^2^) + 5.0469*P*] where *P* = (*F*_o_^2^ + 2*F*_c_^2^)/3
*R*[*F*^2^ > 2σ(*F*^2^)] = 0.016	(Δ/σ)_max_ < 0.001
*wR*(*F*^2^) = 0.038	Δρ_max_ = 0.50 e Å^−^^3^
*S* = 1.14	Δρ_min_ = −0.48 e Å^−^^3^
208 reflections	Extinction correction: *SHELXL*, Fc^*^=kFc[1+0.001xFc^2^λ^3^/sin(2θ)]^-1/4^
15 parameters	Extinction coefficient: 0.00144 (17)
1 restraint	Absolute structure: Flack (1983), 90 Friedel pairs
Primary atom site location: isomorphous structure methods	Flack parameter: 0.01 (4)

### Special details

**Table:**

Geometry. All e.s.d.'s (except the e.s.d. in the dihedral angle between two l.s. planes) are estimated using the full covariance matrix. The cell e.s.d.'s are taken into account individually in the estimation of e.s.d.'s in distances, angles and torsion angles; correlations between e.s.d.'s in cell parameters are only used when they are defined by crystal symmetry. An approximate (isotropic) treatment of cell e.s.d.'s is used for estimating e.s.d.'s involving l.s. planes.
Refinement. Refinement of *F*^2^ against ALL reflections. The weighted *R*-factor *wR* and goodness of fit *S* are based on *F*^2^, conventional *R*-factors *R* are based on *F*, with *F* set to zero for negative *F*^2^. The threshold expression of *F*^2^ > σ(*F*^2^) is used only for calculating *R*-factors(gt) *etc*. and is not relevant to the choice of reflections for refinement. *R*-factors based on *F*^2^ are statistically about twice as large as those based on *F*, and *R*- factors based on ALL data will be even larger.

### Fractional atomic coordinates and isotropic or equivalent isotropic displacement parameters (Å^2^)

**Table:**

	*x*	*y*	*z*	*U*_iso_*/*U*_eq_	
Cs1	0.7500	0.7500	0.7500	0.0599 (3)	
Mg1	0.0000	0.0000	0.0000	0.0250 (6)	
As1	0.2500	0.2500	0.2500	0.0201 (2)	
O1	0.2031 (3)	0.0000	0.0000	0.0508 (8)	
O2	0.34550 (19)	0.34550 (19)	0.34550 (19)	0.0269 (7)	
H1	0.249 (3)	0.045 (2)	0.045 (2)	0.053 (12)*	

### Atomic displacement parameters (Å^2^)

**Table:**

	*U*^11^	*U*^22^	*U*^33^	*U*^12^	*U*^13^	*U*^23^
Cs1	0.0599 (3)	0.0599 (3)	0.0599 (3)	0.000	0.000	0.000
Mg1	0.0250 (6)	0.0250 (6)	0.0250 (6)	0.000	0.000	0.000
As1	0.0201 (2)	0.0201 (2)	0.0201 (2)	0.000	0.000	0.000
O1	0.0259 (13)	0.0632 (13)	0.0632 (13)	0.000	0.000	−0.033 (2)
O2	0.0269 (7)	0.0269 (7)	0.0269 (7)	−0.0036 (7)	−0.0036 (7)	−0.0036 (7)

### Geometric parameters (Å, °)

**Table:**

Cs1—O1^i^	3.6239 (5)	Mg1—O1^xiii^	2.064 (3)
Cs1—O1^ii^	3.6239 (5)	Mg1—O1^xiv^	2.064 (3)
Cs1—O1^iii^	3.6239 (5)	Mg1—O1^xv^	2.064 (3)
Cs1—O1^iv^	3.6239 (5)	Mg1—O1^xvi^	2.064 (3)
Cs1—O1^v^	3.6239 (5)	Mg1—O1^xvii^	2.064 (3)
Cs1—O1^vi^	3.6239 (5)	Mg1—O1	2.064 (3)
Cs1—O1^vii^	3.6239 (5)	As1—O2	1.681 (3)
Cs1—O1^viii^	3.6239 (5)	As1—O2^xviii^	1.681 (3)
Cs1—O1^ix^	3.6239 (5)	As1—O2^xix^	1.681 (3)
Cs1—O1^x^	3.6239 (5)	As1—O2^xx^	1.681 (3)
Cs1—O1^xi^	3.6239 (5)	O1—H1	0.79 (2)
Cs1—O1^xii^	3.6239 (5)		
			
O1^i^—Cs1—O1^ii^	47.49 (8)	O1^iii^—Cs1—O1^xi^	72.12 (8)
O1^i^—Cs1—O1^iii^	47.49 (8)	O1^iv^—Cs1—O1^xi^	47.49 (8)
O1^ii^—Cs1—O1^iii^	47.49 (8)	O1^v^—Cs1—O1^xi^	90.991 (13)
O1^i^—Cs1—O1^iv^	164.89 (10)	O1^vi^—Cs1—O1^xi^	119.429 (8)
O1^ii^—Cs1—O1^iv^	119.430 (7)	O1^vii^—Cs1—O1^xi^	72.12 (8)
O1^iii^—Cs1—O1^iv^	119.429 (7)	O1^viii^—Cs1—O1^xi^	119.429 (7)
O1^i^—Cs1—O1^v^	119.429 (8)	O1^ix^—Cs1—O1^xi^	47.49 (8)
O1^ii^—Cs1—O1^v^	164.89 (10)	O1^x^—Cs1—O1^xi^	164.89 (10)
O1^iii^—Cs1—O1^v^	119.429 (7)	O1^i^—Cs1—O1^xii^	72.12 (8)
O1^iv^—Cs1—O1^v^	72.12 (8)	O1^ii^—Cs1—O1^xii^	119.429 (7)
O1^i^—Cs1—O1^vi^	119.429 (8)	O1^iii^—Cs1—O1^xii^	90.991 (13)
O1^ii^—Cs1—O1^vi^	119.429 (8)	O1^iv^—Cs1—O1^xii^	119.429 (8)
O1^iii^—Cs1—O1^vi^	164.89 (10)	O1^v^—Cs1—O1^xii^	47.49 (8)
O1^iv^—Cs1—O1^vi^	72.12 (8)	O1^vi^—Cs1—O1^xii^	90.991 (13)
O1^v^—Cs1—O1^vi^	72.12 (8)	O1^vii^—Cs1—O1^xii^	47.49 (8)
O1^i^—Cs1—O1^vii^	90.991 (13)	O1^viii^—Cs1—O1^xii^	119.429 (7)
O1^ii^—Cs1—O1^vii^	119.430 (7)	O1^ix^—Cs1—O1^xii^	164.89 (10)
O1^iii^—Cs1—O1^vii^	72.12 (8)	O1^x^—Cs1—O1^xii^	72.12 (8)
O1^iv^—Cs1—O1^vii^	90.991 (13)	O1^xi^—Cs1—O1^xii^	119.429 (8)
O1^v^—Cs1—O1^vii^	47.49 (8)	O1^xiii^—Mg1—O1^xiv^	180.0
O1^vi^—Cs1—O1^vii^	119.429 (8)	O1^xiii^—Mg1—O1^xv^	90.0
O1^i^—Cs1—O1^viii^	90.991 (13)	O1^xiv^—Mg1—O1^xv^	90.0
O1^ii^—Cs1—O1^viii^	72.12 (8)	O1^xiii^—Mg1—O1^xvi^	90.0
O1^iii^—Cs1—O1^viii^	119.430 (7)	O1^xiv^—Mg1—O1^xvi^	90.0
O1^iv^—Cs1—O1^viii^	90.991 (13)	O1^xv^—Mg1—O1^xvi^	180.0
O1^v^—Cs1—O1^viii^	119.429 (8)	O1^xiii^—Mg1—O1^xvii^	90.0
O1^vi^—Cs1—O1^viii^	47.49 (8)	O1^xiv^—Mg1—O1^xvii^	90.0
O1^vii^—Cs1—O1^viii^	164.89 (10)	O1^xv^—Mg1—O1^xvii^	90.0
O1^i^—Cs1—O1^ix^	119.429 (7)	O1^xvi^—Mg1—O1^xvii^	90.0
O1^ii^—Cs1—O1^ix^	72.12 (8)	O1^xiii^—Mg1—O1	90.0
O1^iii^—Cs1—O1^ix^	90.991 (13)	O1^xiv^—Mg1—O1	90.0
O1^iv^—Cs1—O1^ix^	47.49 (8)	O1^xv^—Mg1—O1	90.0
O1^v^—Cs1—O1^ix^	119.429 (8)	O1^xvi^—Mg1—O1	90.0
O1^vi^—Cs1—O1^ix^	90.991 (13)	O1^xvii^—Mg1—O1	180.0
O1^vii^—Cs1—O1^ix^	119.429 (8)	O2—As1—O2^xviii^	109.5
O1^viii^—Cs1—O1^ix^	72.12 (8)	O2—As1—O2^xix^	109.471 (1)
O1^i^—Cs1—O1^x^	72.12 (8)	O2^xviii^—As1—O2^xix^	109.5
O1^ii^—Cs1—O1^x^	90.991 (13)	O2—As1—O2^xx^	109.5
O1^iii^—Cs1—O1^x^	119.430 (7)	O2^xviii^—As1—O2^xx^	109.5
O1^iv^—Cs1—O1^x^	119.429 (8)	O2^xix^—As1—O2^xx^	109.5
O1^v^—Cs1—O1^x^	90.991 (13)	Mg1—O1—Cs1^xxi^	97.56 (5)
O1^vi^—Cs1—O1^x^	47.49 (8)	Mg1—O1—Cs1^xxii^	97.56 (5)
O1^vii^—Cs1—O1^x^	119.429 (7)	Cs1^xxi^—O1—Cs1^xxii^	164.89 (10)
O1^viii^—Cs1—O1^x^	47.49 (8)	Mg1—O1—H1	126 (3)
O1^ix^—Cs1—O1^x^	119.429 (7)	Cs1^xxi^—O1—H1	85.6 (3)
O1^i^—Cs1—O1^xi^	119.430 (7)	Cs1^xxii^—O1—H1	85.6 (3)
O1^ii^—Cs1—O1^xi^	90.991 (13)		

Symmetry codes: (i) −*y*+1, *z*+1, −*x*+1; (ii) *z*+1, −*x*+1, −*y*+1; (iii) −*x*+1, −*y*+1, *z*+1; (iv) −*y*+1/2, *z*+1/2, −*x*+1; (v) *z*+1/2, −*x*+1, −*y*+1/2; (vi) −*x*+1, −*y*+1/2, *z*+1/2; (vii) *y*+1/2, *z*+1, *x*+1/2; (viii) *y*+1, *z*+1/2, *x*+1/2; (ix) *x*+1/2, *y*+1/2, *z*+1; (x) *z*+1, *x*+1/2, *y*+1/2; (xi) *z*+1/2, *x*+1/2, *y*+1; (xii) *x*+1/2, *y*+1, *z*+1/2; (xiii) −*y*, *z*, −*x*; (xiv) *y*, *z*, *x*; (xv) *z*, −*x*, −*y*; (xvi) *z*, *x*, *y*; (xvii) −*x*, −*y*, *z*; (xviii) −*x*+1/2, *y*, −*z*+1/2; (xix) *x*, −*y*+1/2, −*z*+1/2; (xx) −*x*+1/2, −*y*+1/2, *z*; (xxi) *x*−1/2, *y*−1/2, *z*−1; (xxii) *x*−1/2, *y*−1, *z*−1/2.

### Hydrogen-bond geometry (Å, °)

**Table:**

*D*—H···*A*	*D*—H	H···*A*	*D*···*A*	*D*—H···*A*
O1—H1···O2^xix^	0.79 (2)	1.86 (2)	2.650 (2)	176 (4)

Symmetry codes: (xix) *x*, −*y*+1/2, −*z*+1/2.
